# Supplementary material for: The Relationship between Burrow Opening Dimensions and Biomass of Intertidal Macroinvertebrates by Feeding Mode (Surface Deposit Feeders vs. Suspension Feeders)
Source: Animals (Basel). 2022 Oct 21;12(20):2878. doi: 10.3390/ani12202878 (PMC9597845; doi:10.3390/ani12202878)

Macrophthalmus japonicus (De Haan)

The *Macrophthalmus japonicus* is one of the most common crabs found in the tidal flats in Korea. It is taxonomically a species of the family *Ocypodidae*. It is used for food and it is also frequently used as fishing bait. In relation to its overall black appearance, its Korean name, chilgae was derived from chik, the Korean word for the arrowroot (*Pueraria thunbergiana*).
This is one of the very common species that can be seen in all the tidal flats with mud. It is a typical deposit–feeder that eats sediments using its claw like a spoon. During the nighttime and during seawater submergence, the crabs of this species stay inside the burrows. When the water has drained away from the burrow, their first activity is to repair the inside of the burrow, and then they start to do feeding activity again. In summer, in the middle of the day, they sometimes enjoy sunbathing around the burrows spreading their claws without moving. They continue this behavior for a maximum of two hours, which is presumed to be an act for removing the parasites by drying the body. The burrows have one or two entrances. The form of the entrance is close to an ellipse, and it extends obliquely inside the sediments. The overall shape of the burrows is a "U"–shaped structure with a branched gallery or a "J"–shaped structure. The depth of the burrow is less than 30 cm, but the total length may exceed 1 m.

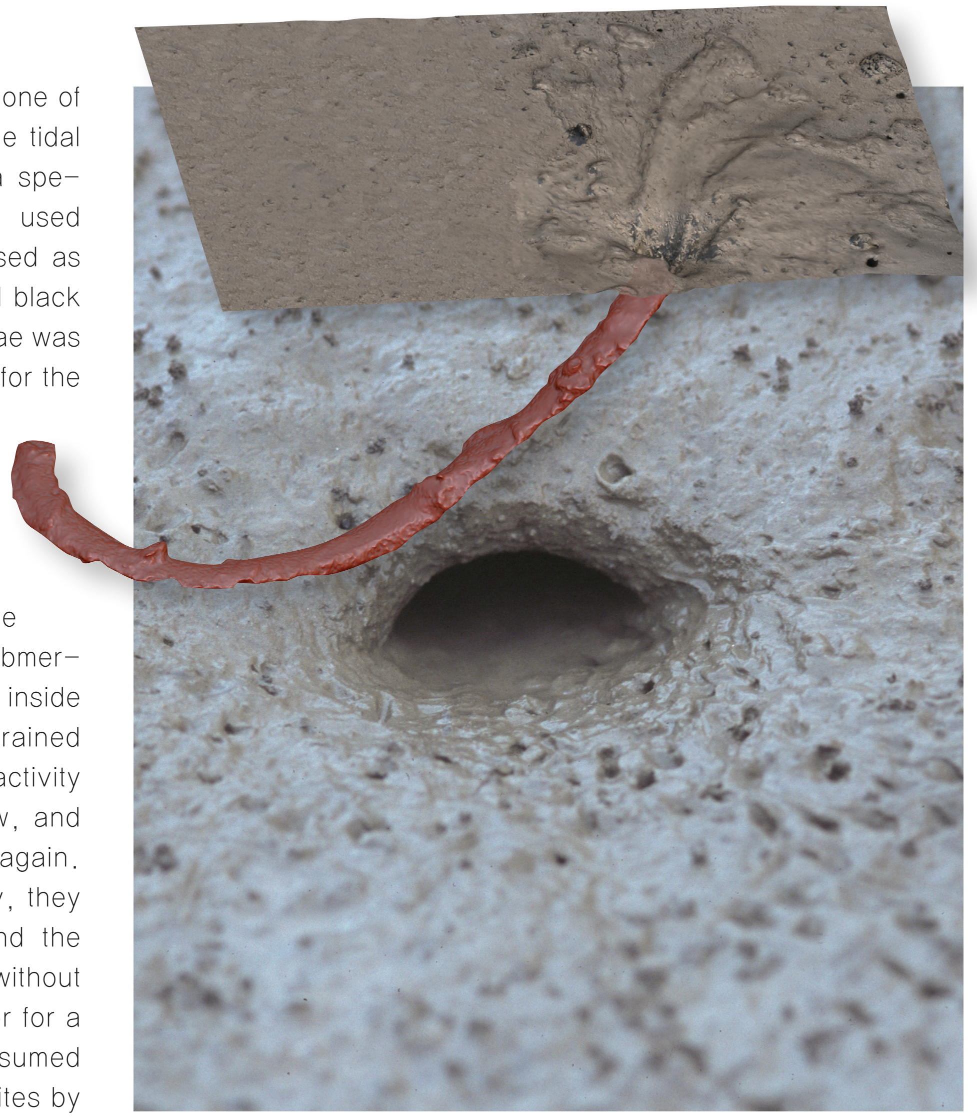

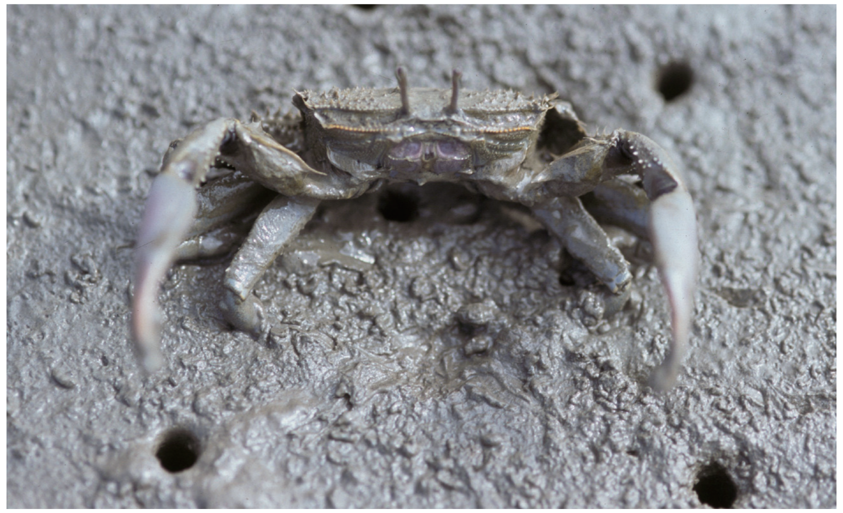

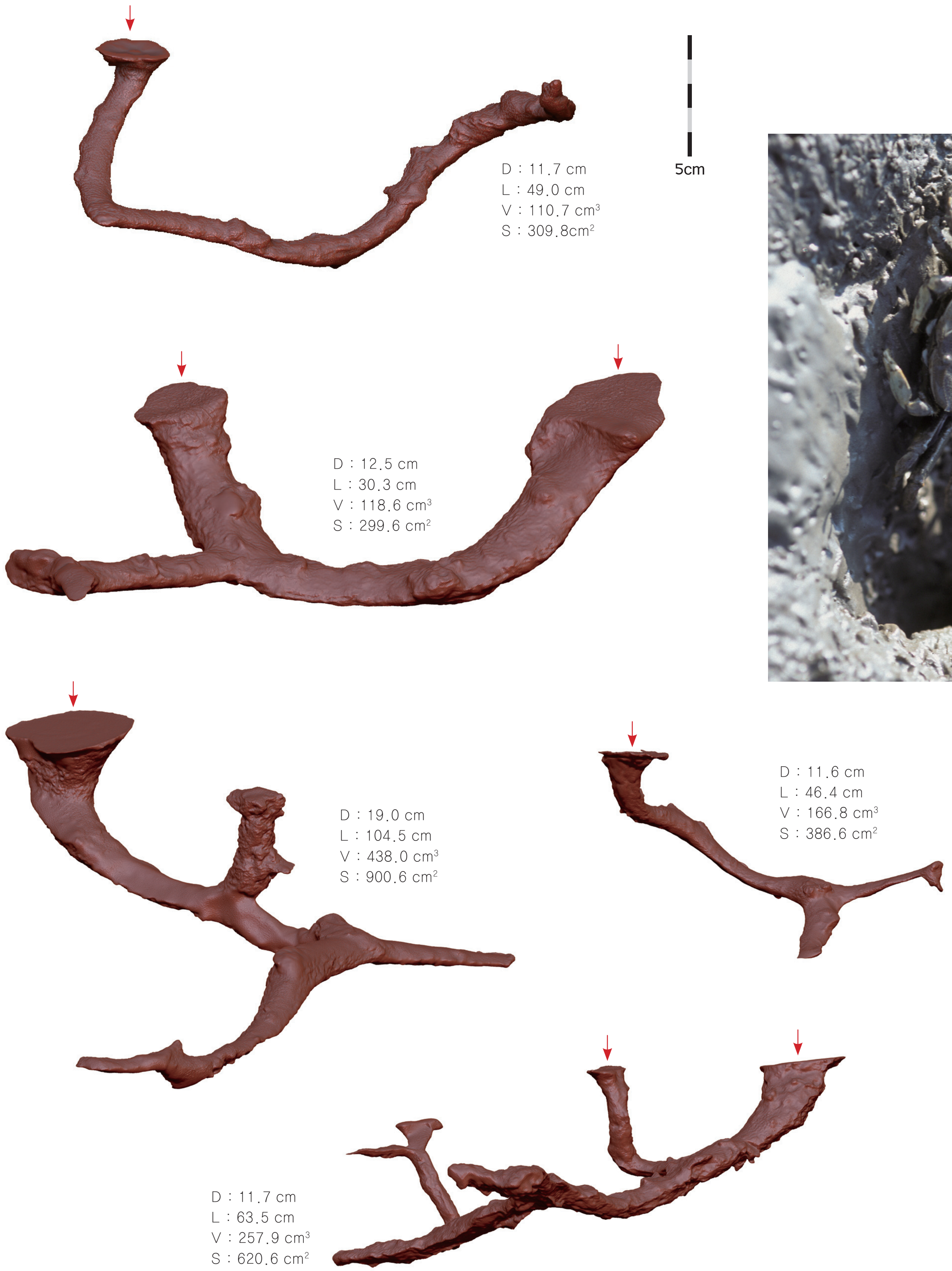

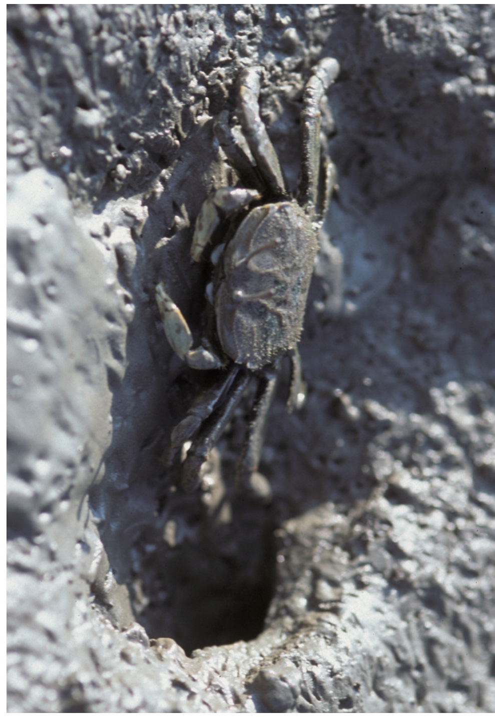

Uca arcuata (De Haan)

The *Uca arcuata*, often called bulgeunbal nongge in Korea, a species of Family Ocypodidae. A feature of its appearance is that one claw of males is large. There are only two species of nongge reported in Korea, along with hinbal nongge (*Uca lactea*). it was named nongge in Korea because it has a dark red appearance. There are more than 100 species of nongge (the genus Uca) in the world known to date, and in general, they are collectively called "fiddler crabs." The word 'fiddler' means a violinist. Its name was derived from the fact that when it shakes the big claw up and down, it looks like it is playing a violin. 'Nongge' lives in groups in the areas near salt marshes close to the land or in the places with a high mud content around the tidal flats. Especially, it shows patch distributions along the dry areas avoiding the environments where water is accumulated on the tidal flat. It eats sediments using craws, but the claws of males are not used for feeding activity. The big claws are used to fight against enemies or attract females.

At the entrance of the burrow, there is a fence with a height of 1 to 2 cm along the perimeter. Fences are common in the burrows of females, but not in those of males. The fence is formed by collecting and piling up the surrounding soil with the claws, and it is thought that males do not make the fence because it is difficult to handle the soil with the big claws. Instead, males use the big claws when they feel stressed or when water flows in, cutting the sediments around the burrows into rounded, flat shapes to block the entrance of the burrows. The burrow of this species has one entrance, and it extends downwards in a straight or slightly curved form. The diameter of the entrance is about 3cm, and the maximum length of the burrow is 1m and the maximum depth is 65cm. The size of the burrow varies according to the size of the individual, and in the burrows examined so far, the total volume of an burrow is up to 568 cm^3.

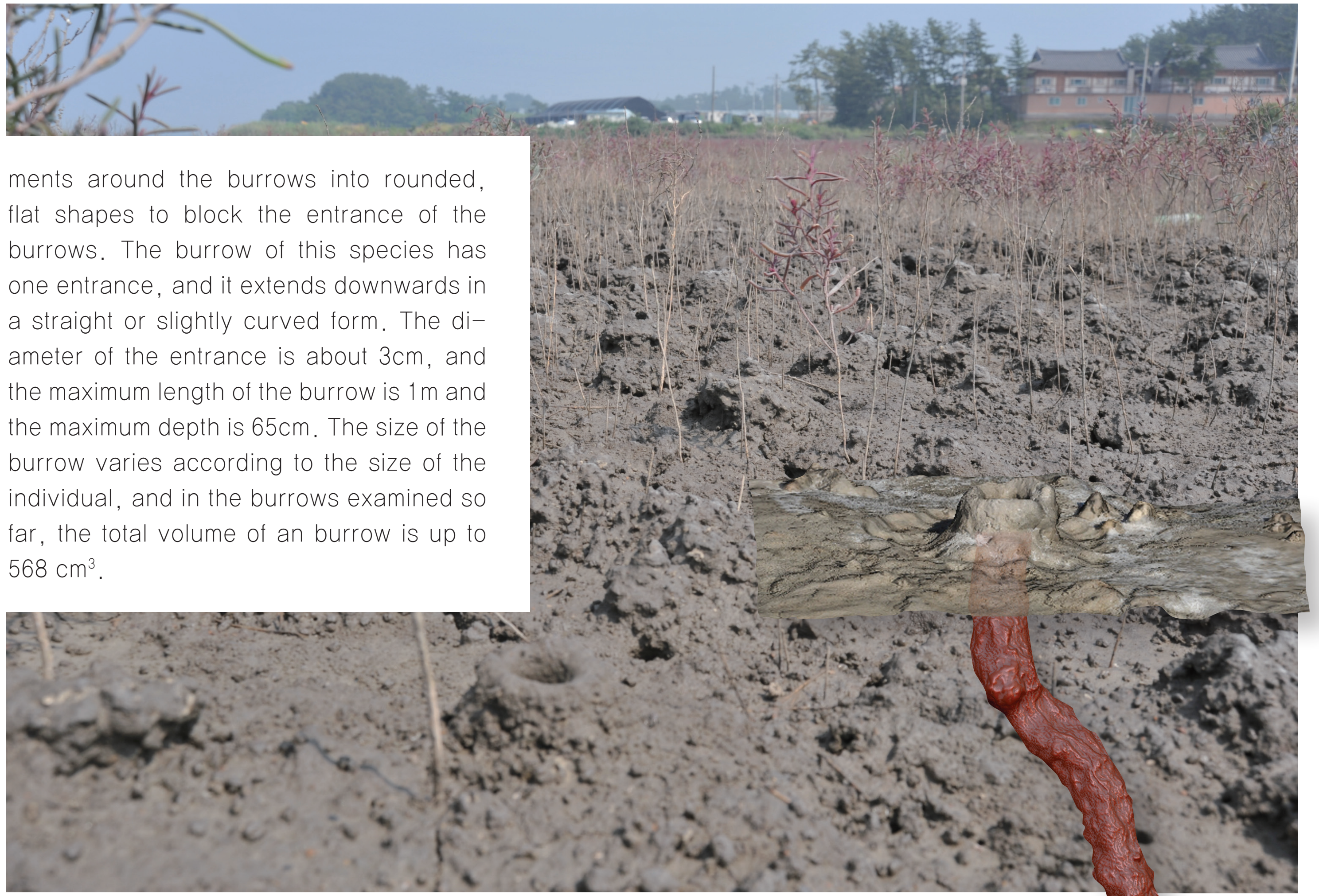

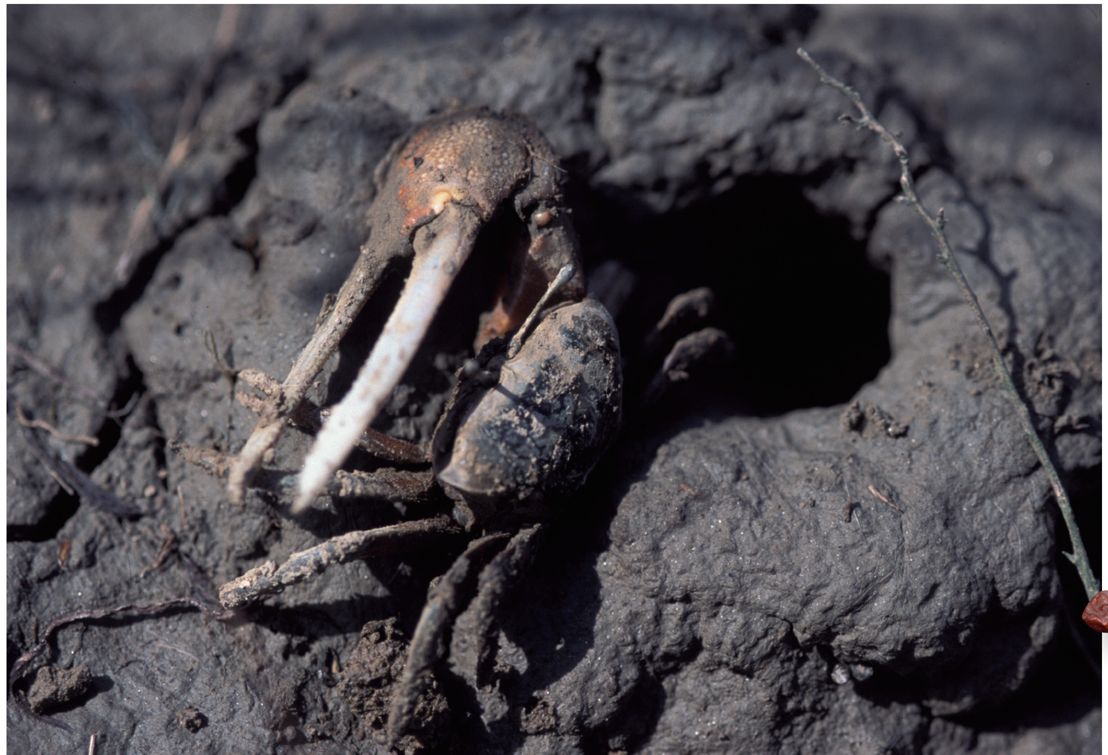

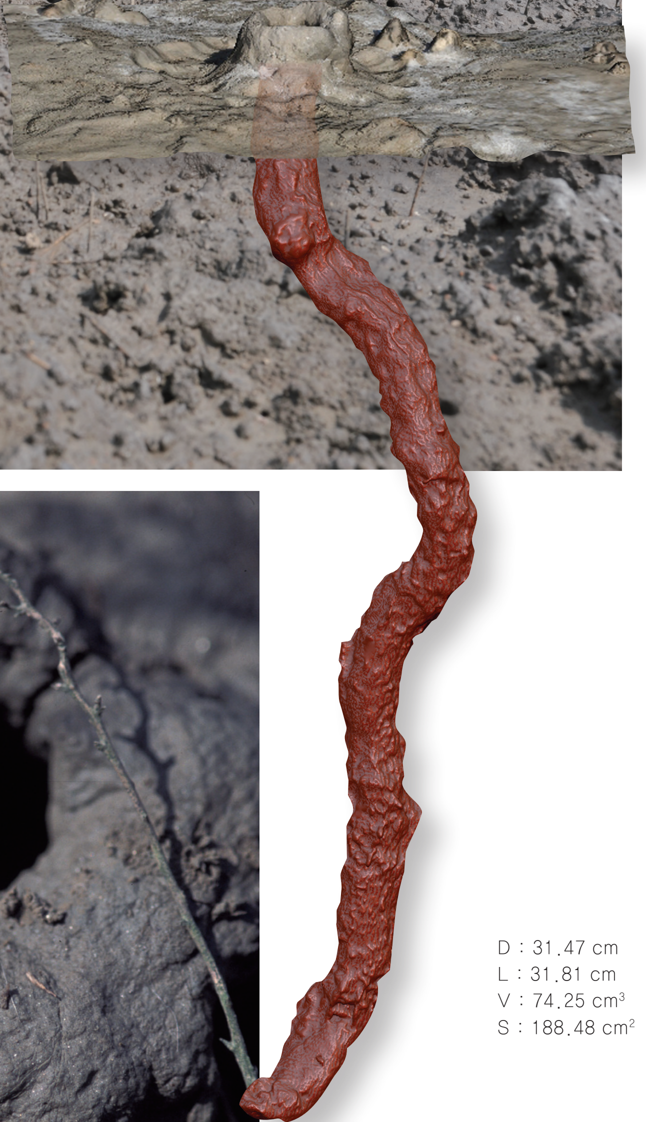

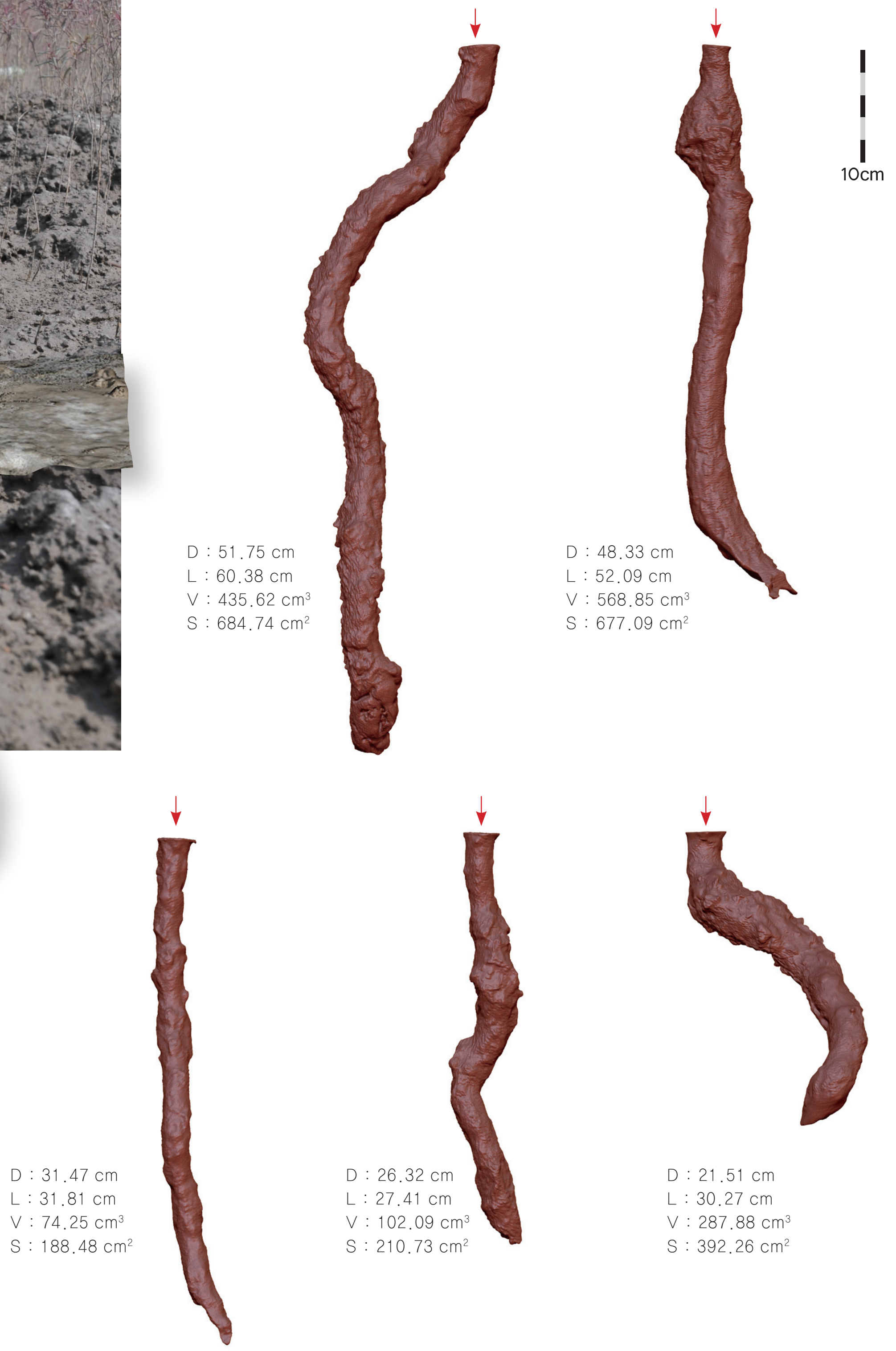

Uca lactea lactea (De Haan)

In contrast to the *Uca arcuata*, the *Uca lactea lactea* has a single white claw, and it is a species of the family *Ocypodidae*. This species is among the crabs designated as the endangered species among the crabs inhabiting the tidal flats, along with the *Pseudohelice subquadrata*, *Chasmagnathus convexus*, and *Sesarmops intermedius*. The claw of females are not large compared to that of males, and males have a large left or right claw. The same can be said of the *Uca arcuata*. This species mainly inhabits the places where the sand content is higher among the areas with mixed mud and sand at the upper part of the tidal flats. However, it is not observed in the sediments composed of only sand such as the seashore. At the entrance of the burrow, there are often clumps of soil that surround the entrance like a fence, but in some cases, there is not such a fence. The burrows of females are more likely to be surrounded by a fence. The shape of the burrow is slightly curved in the middle part, and in some cases, the lower part of the burrow is extended. The depth of the burrow does not exceed 20 cm.

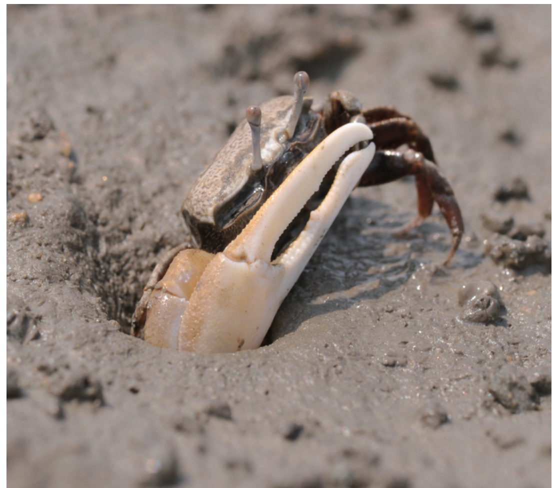

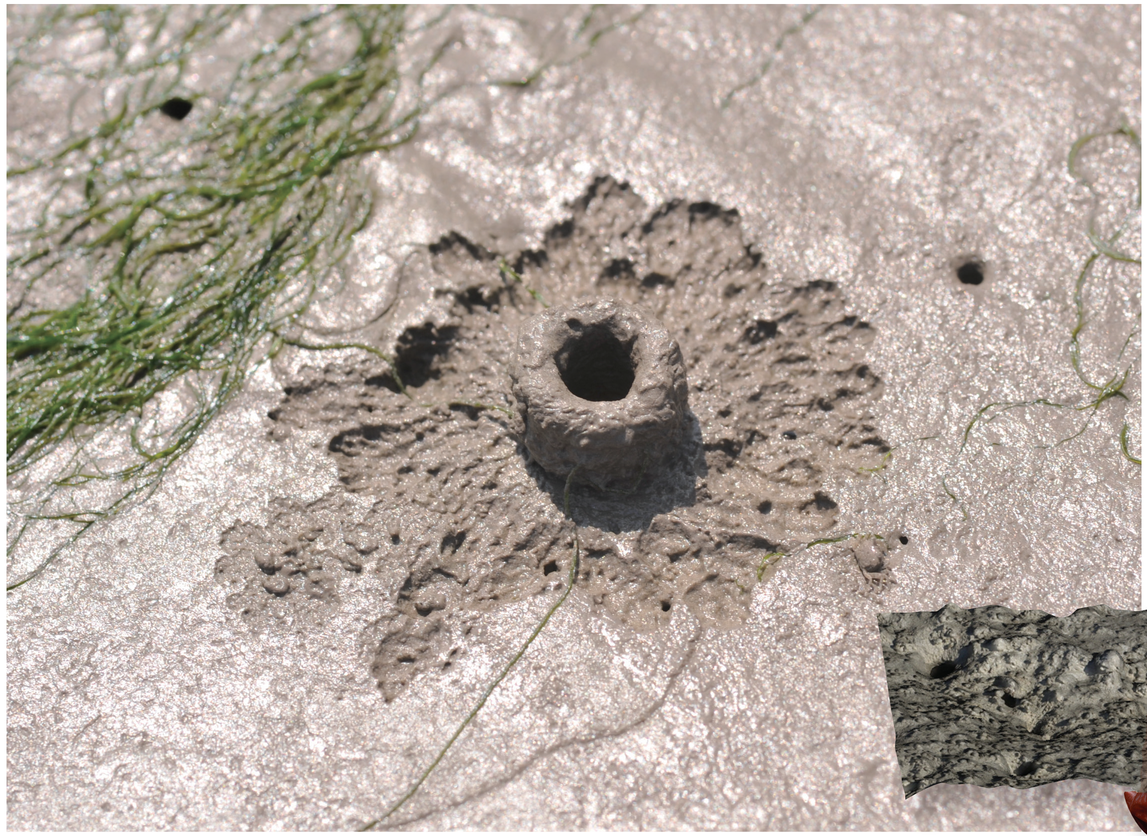

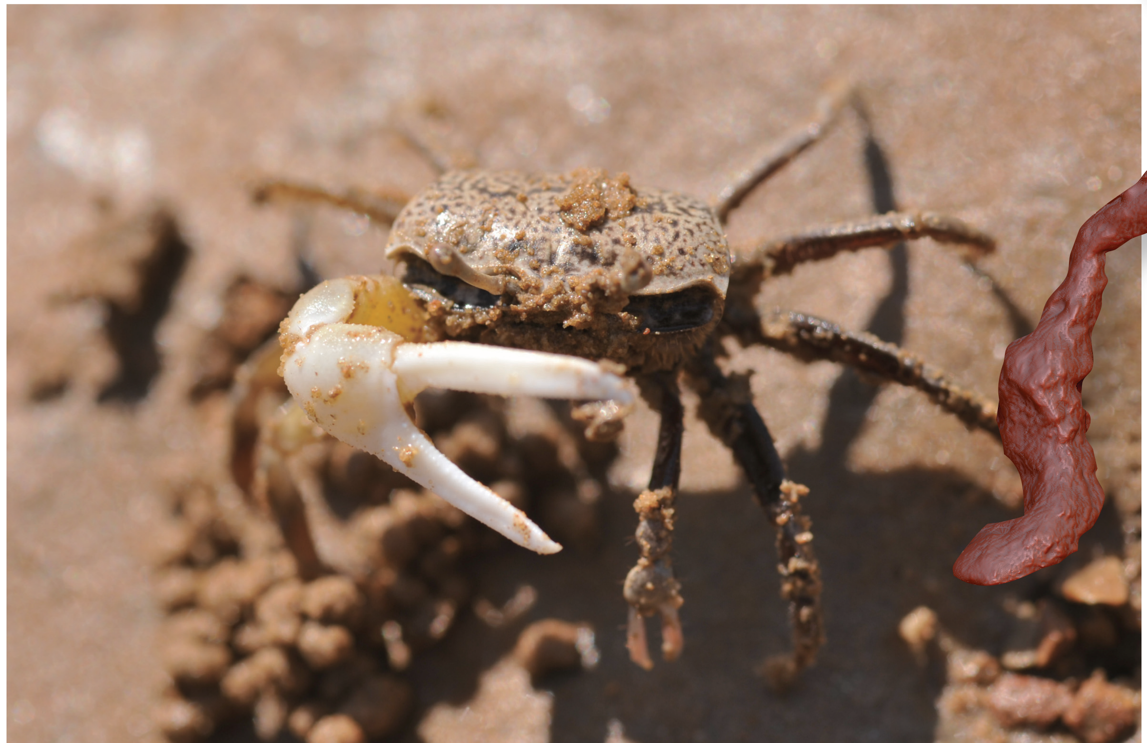

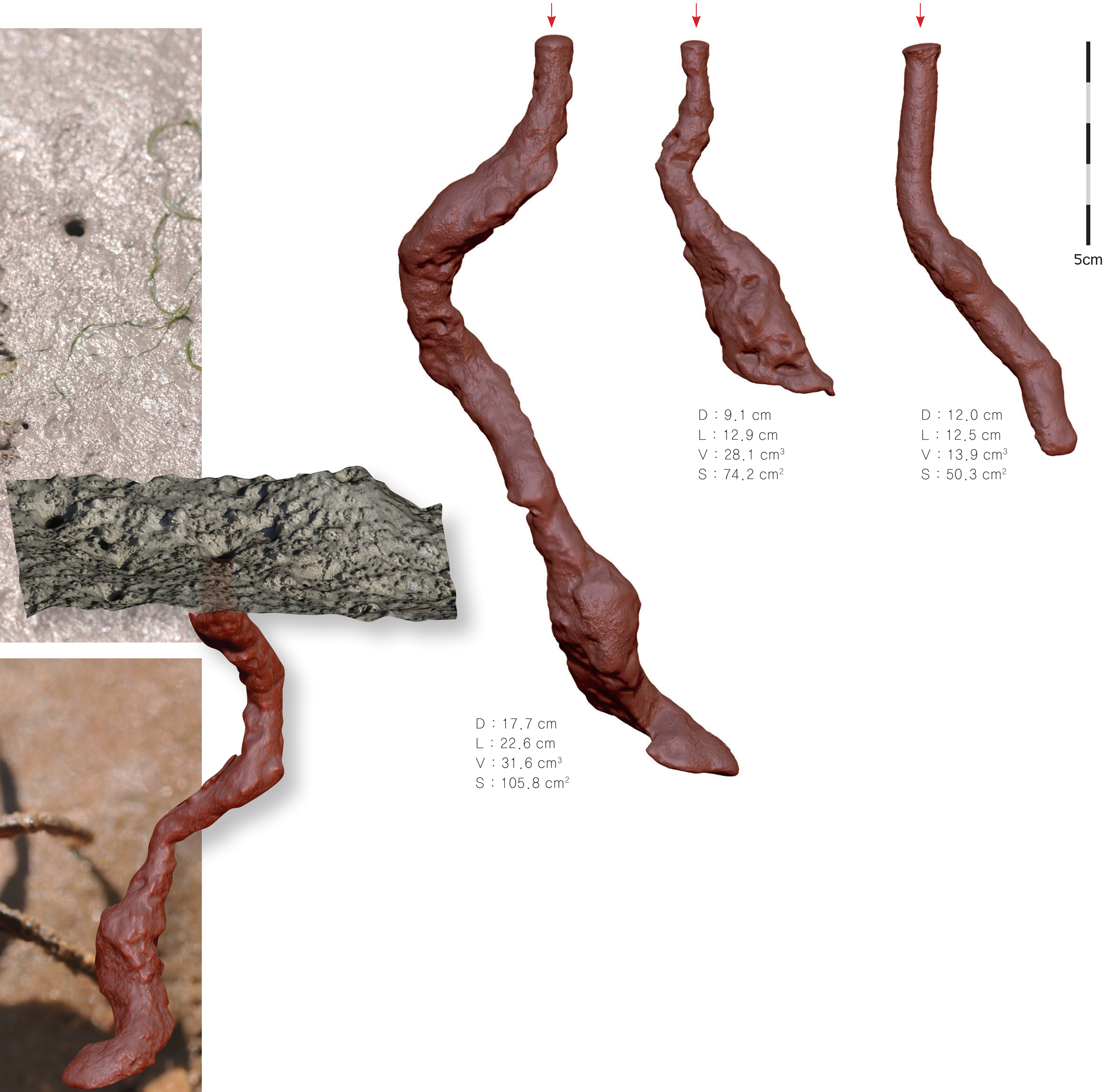

Ocypode stimpsoni Ortmann

The *Ocypode stimpsoni* is a species of the family *Ocypodidae*, and it is called the ghost crab. It digs a large hole in the top-most area of sandy tidal flats and lives in it. It is not well observed during the daytime, and it usually comes out of the burrow at night. It is called the ghost crab because it is mainly active at night. This species is called dallangge in Korea due to the fact that if you listen to it moving over the sandy beach, the noise made by its movements sounds like *dallang-dallang*. It is not used for food in Korea, but in Southeast Asian countries such as Vietnam, it is sometimes caught for food. It mainly eats sediments, takes organic matter in it, and spits clumps made of sediments with a shape of a rice ball cake around the burrow. They also eat dead bodies of fishes or other organisms. The burrow is connected to the surface of the tidal flat through a single entrance and has an "L"-shaped structure that extends vertically downward and is inclined from the middle part. The entrance of the burrow is up to 6.7 cm in diameter, and the passage extends to a depth of 35 cm. The diameter of the widest part of the burrow is about 5 cm, and the total volume of the burrow is up to 705 cm^3.

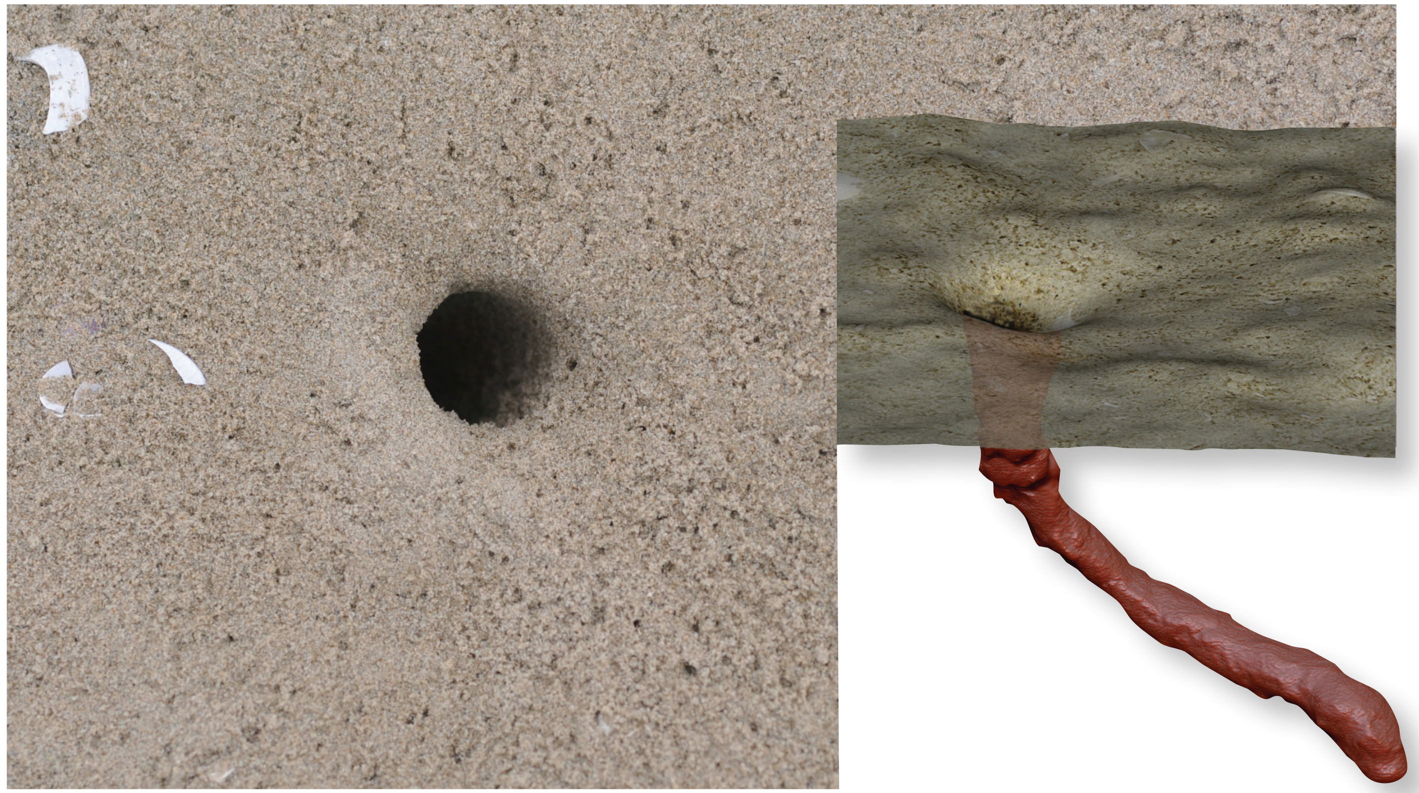

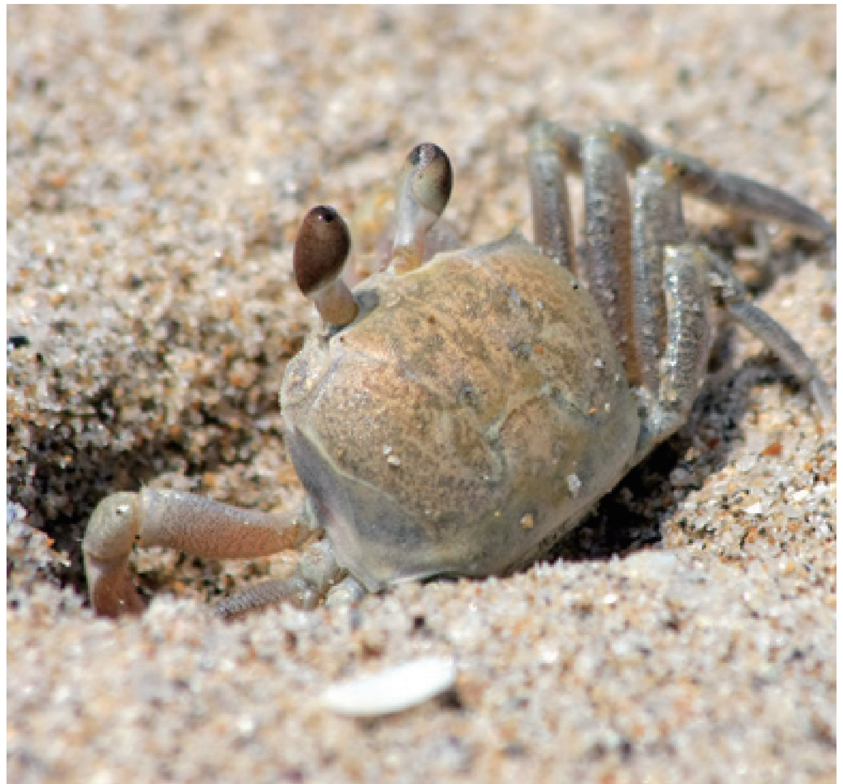

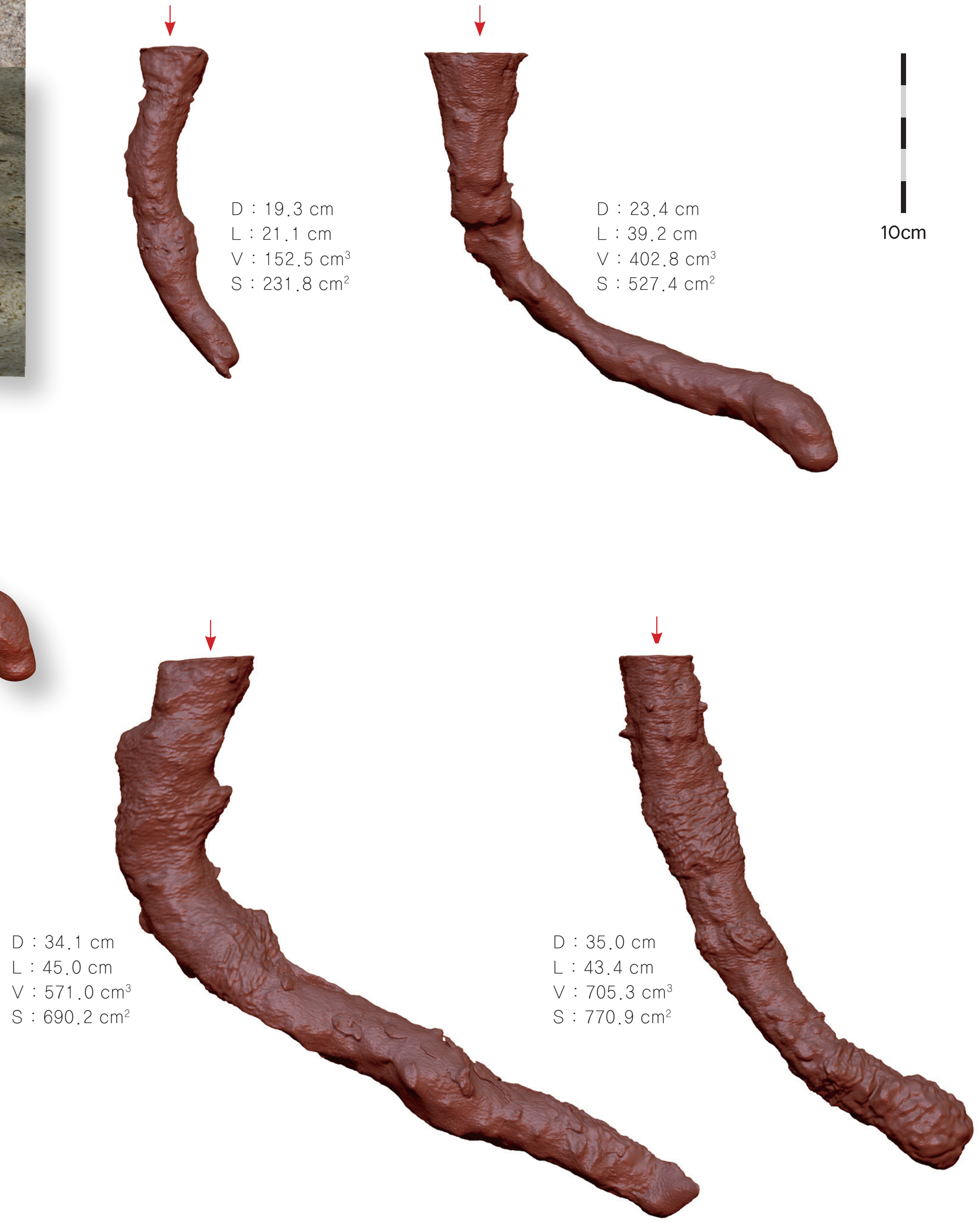

Upogebia major (De Haan)

The *Upogebia major* is a species of the family *Callianassidae*, and it is a species of the order *Decapoda* which crabs belong to. In terms of systematic taxonomy, it has a closer relationship with the Anomura than crabs. It is said that it was named ssok in Korea because it slips away easily from the burrow when fishermen try to catch it ('ssok' is a Korean adverb which expresses the manner of slipping away easily from something). This species has a wide distribution in the middle and lower parts of the tidal flats with a high mud content or with suitably mixed mud and sand. Its burrow is "Y"-shaped, and the inner wall of the burrow is thickly coated with mucous material. It causes water currents in the burrow by moving the pleopods, which are appendages, and at this time, it eats organic matter including plankton in the seawater flowing into the burrow. The burrow has a Y-shaped structure in which two vertical passages connected in the form of a "U" form the upper part and there is a straight passage in the lower part. It may also be branched at a short length from the passage. Compared with the vertical passage, the diameter of the straight passage is slightly wider (vertical passage: 2.8 cm; straight-line passage: 3.2 cm), and the total length of the burrow is up to about 2 m. The depth is often more than 1 m, and the total size may exceed 1,400 cm^3 in volume.

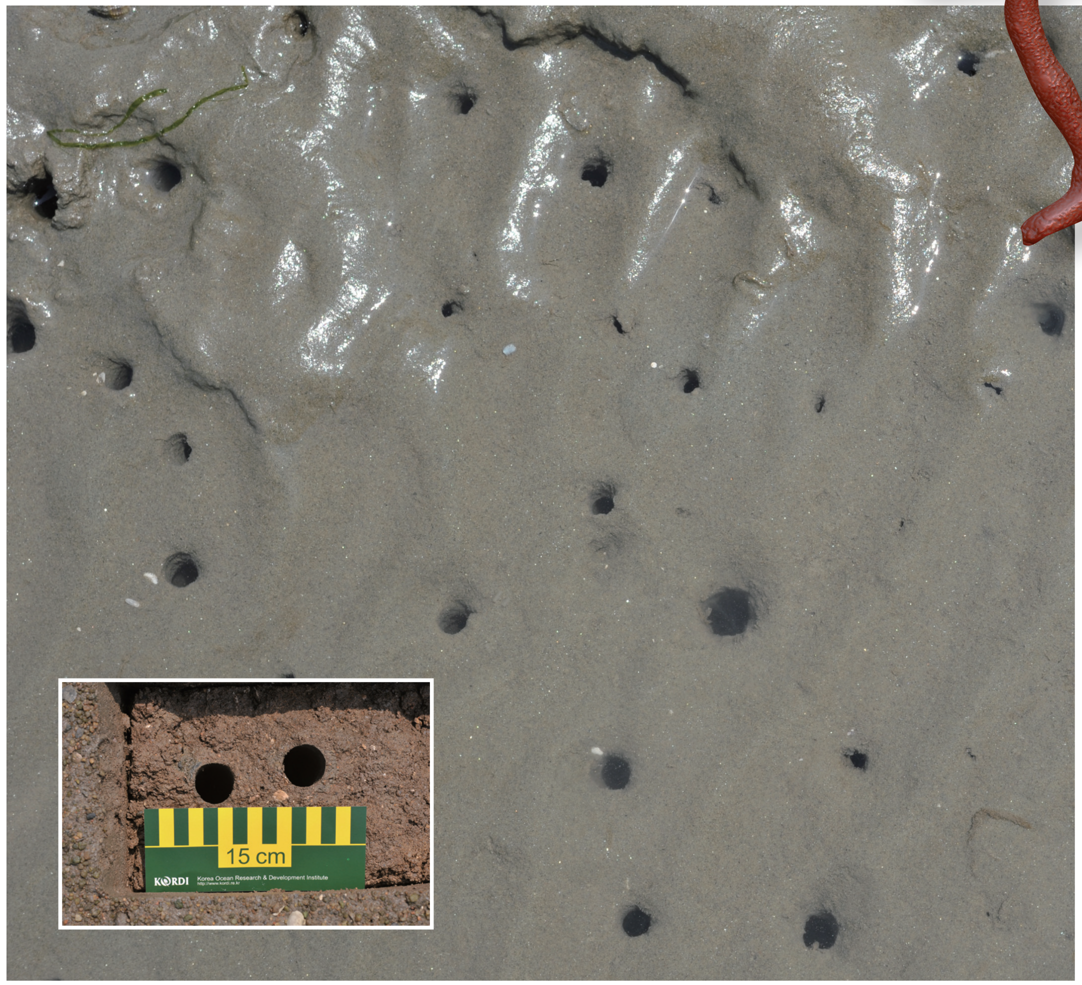

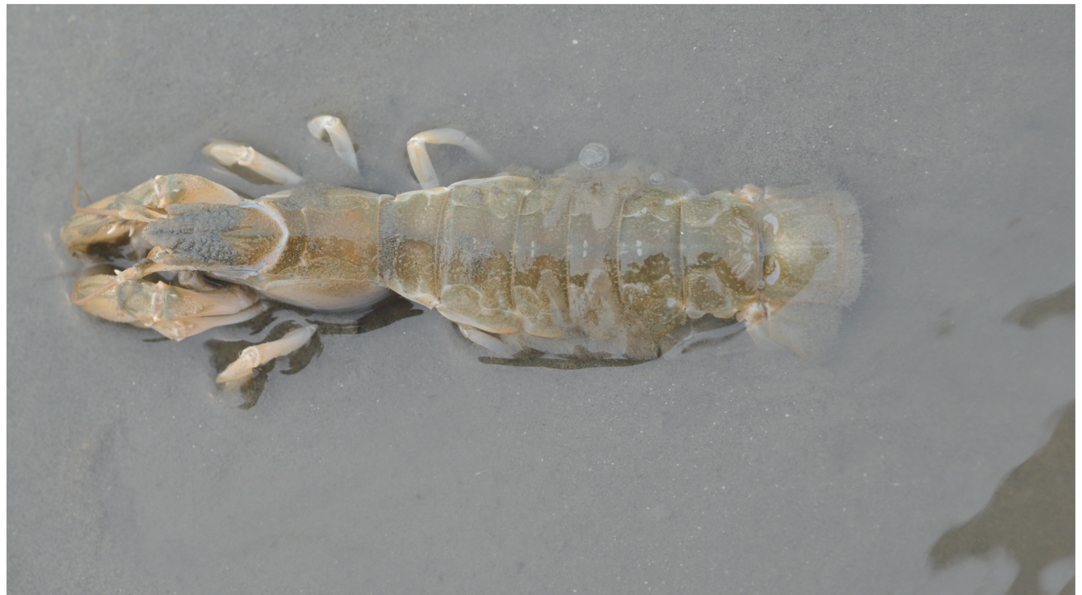

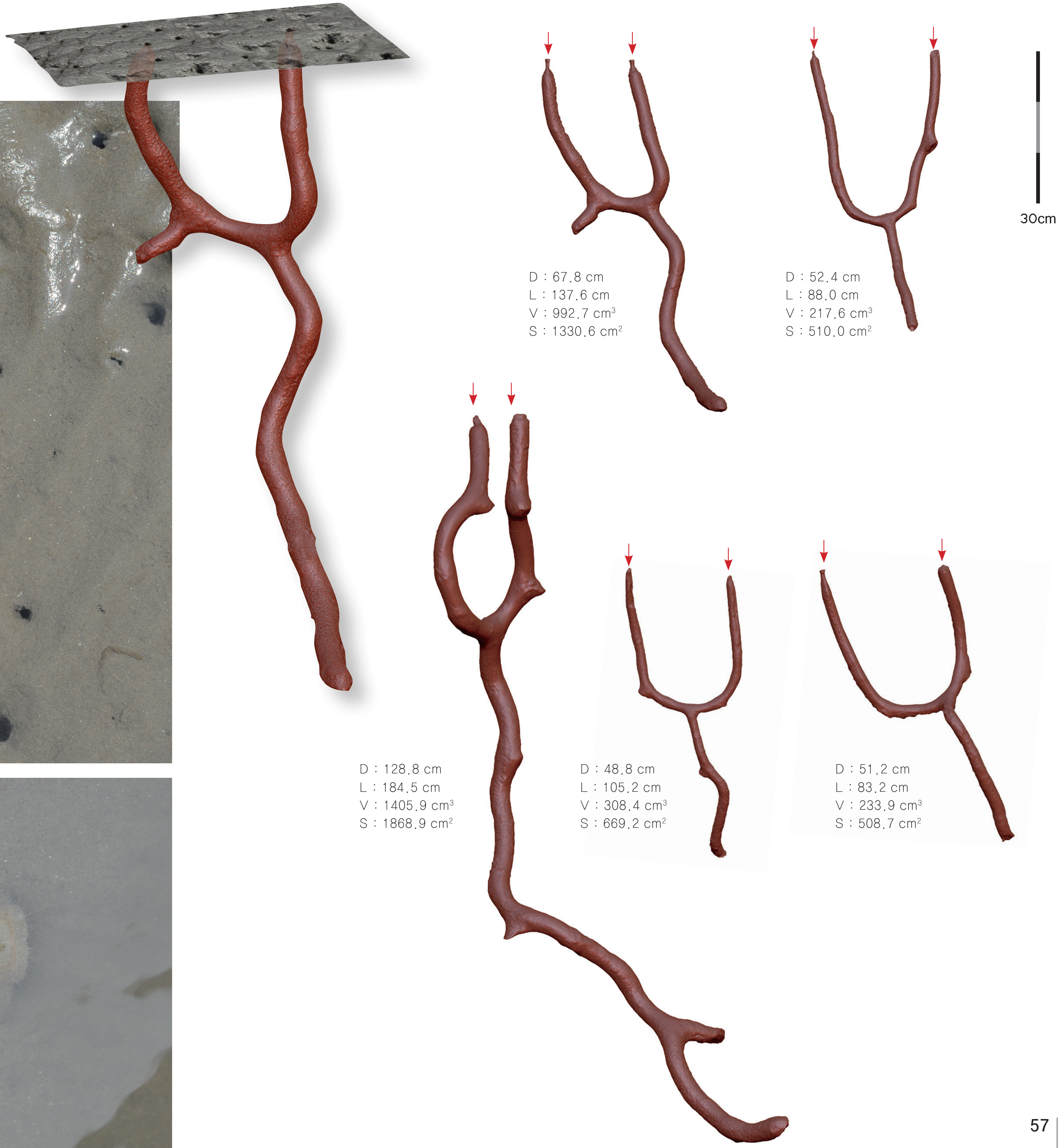

Urechis unicinctus (von Drache)

The *Urechis unicinctus*, a species of the phylum *Echiura*, is a representative food animal produced in the tidal flats. It mostly lives in sediments with a high sand content in the lower part of the tidal flat (near the ebb-tide line), but it also lives in the shallow subtidal zone. The burrow is a "U"-shaped structure with a depth of more than 40 cm. The outer wall of the burrow is coated with a mucous material, and the entrance of the burrow has a shape similar to an upside-down small bowl. There are scattered excreta with an elongated granular form around it. The entrances of the burrow on both sides are vertically downward, then run parallel, and are connected with each other. The diameter of the entrance is about 1.6 cm, which is much narrower than the diameter of the body, but the diameter of the passages running parallel is as wide as 3 cm. According to the analysis data of the burrow of an individual, the length of the burrow reached about 1 m, the surface area and the volume were 684 cm^2 and 1,073 cm^3, respectively.

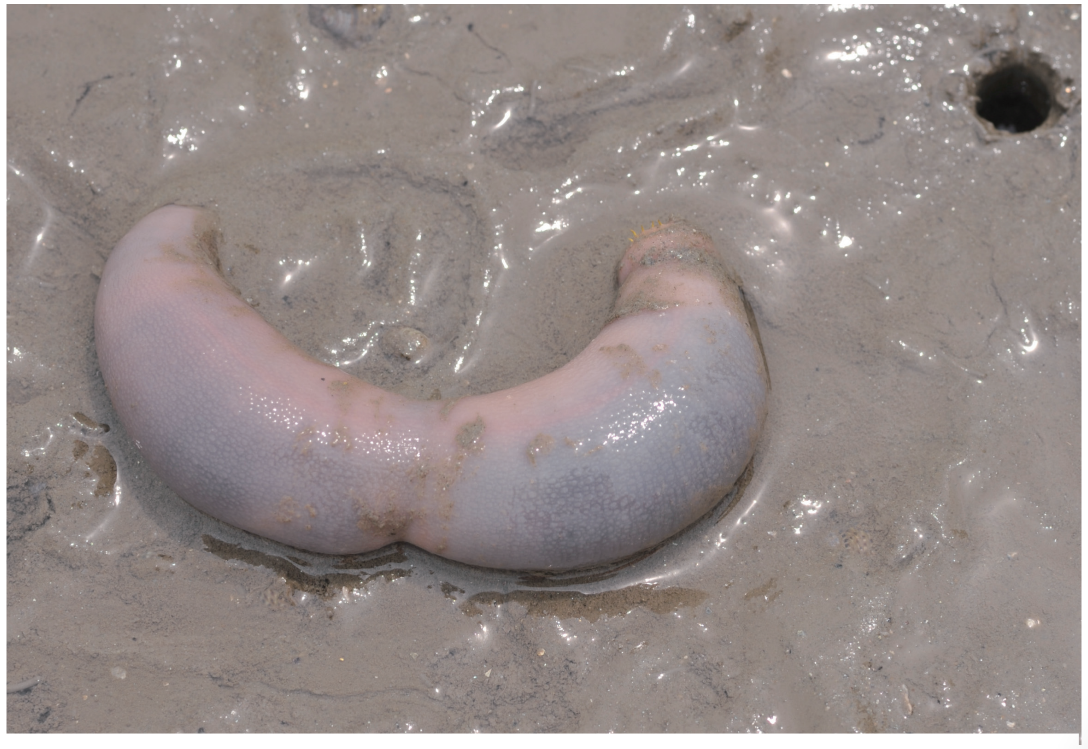

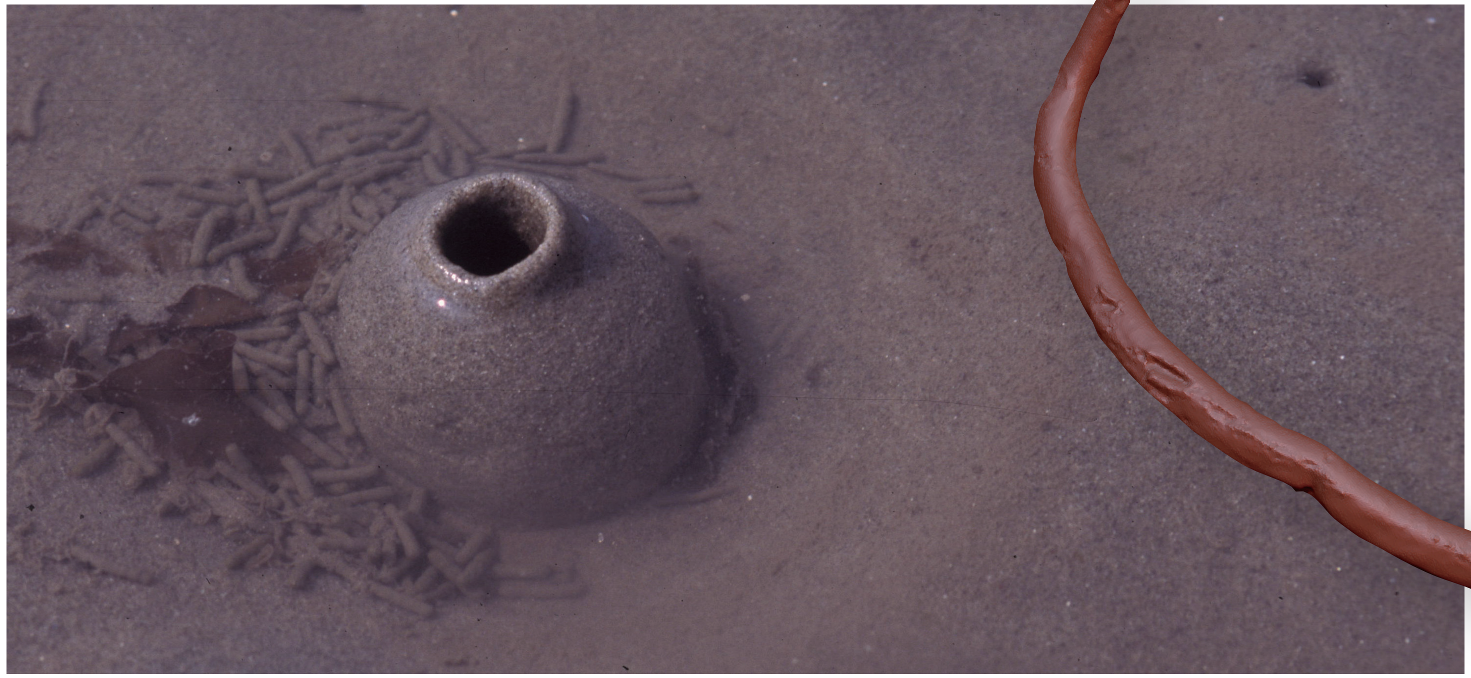

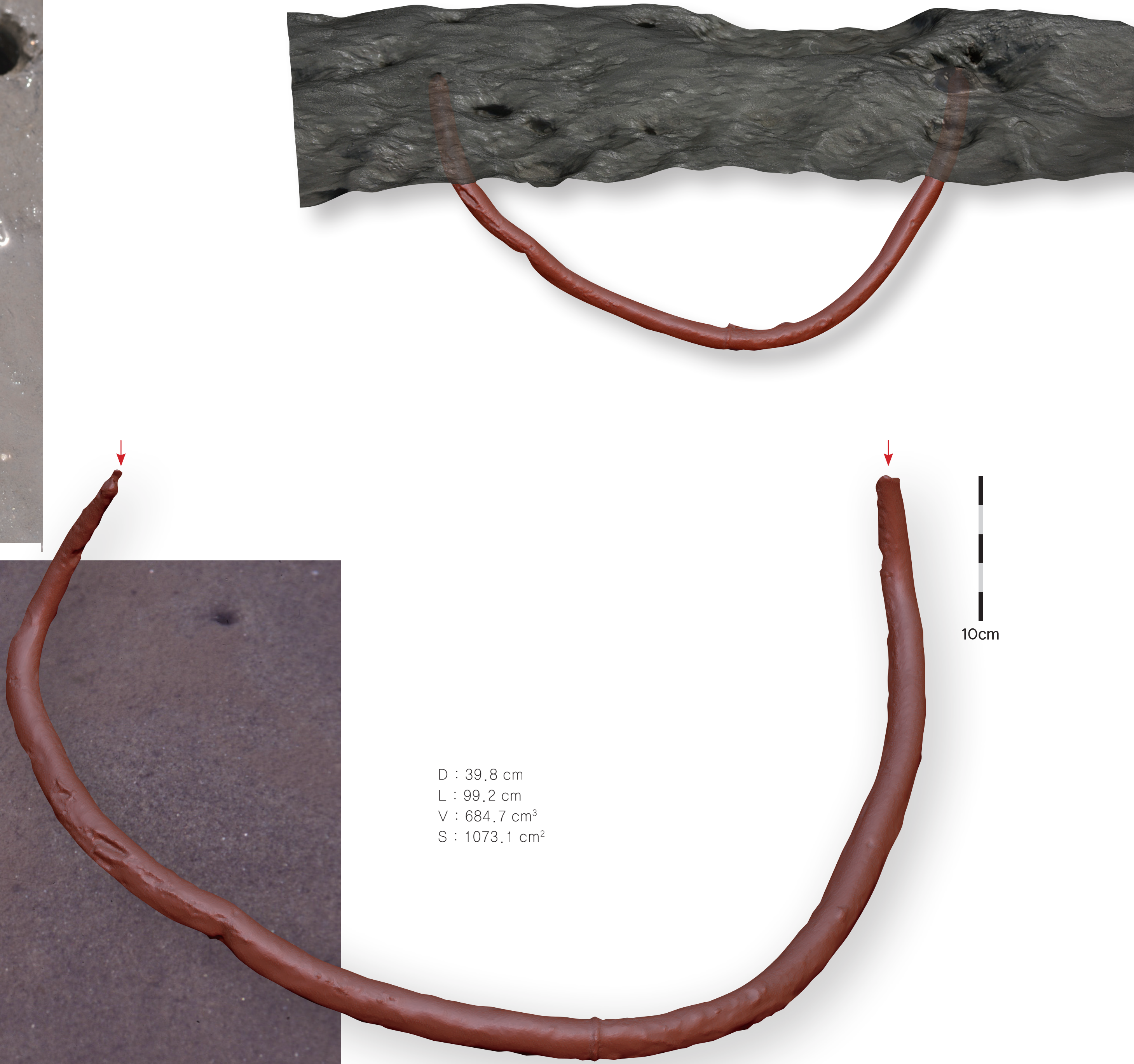

Supplement: Supplementary file 1 [file animals-12-02878-s001.zip › animals-1975821-supplementary.pdf]
